# Mechanistic Insights into the Bornyl Diphosphate Synthase from *Lavandula angustifolia*

**DOI:** 10.3390/cimb47070517

**Published:** 2025-07-04

**Authors:** Dafeng Liu, Na Li, Feng Yu, Yanyan Du, Hongjun Song, Wenshuang Yao

**Affiliations:** 1 Xinjiang Key Laboratory of Lavender Conservation and Utilization, College of Biological Sciences and Technology, Yili Normal University, Yining 835000, China; 2 School of Life Sciences, Xiamen University, Xiamen 361102, China

**Keywords:** *Lavandula angustifolia*, bornyl diphosphate synthase, geranyl diphosphate, enzymatic activity assays, gene expression profiles

## Abstract

Lavender species hold substantial economic importance due to their widespread cultivation for essential oils (EOs). Lavender EOs contain terpenes essential for industries such as cosmetics, personal care, and pharmaceuticals. In the biosynthetic pathway of EOs, *Lavandula angustifolia* bornyl diphosphate synthase (LaBPPS) catalyzes the conversion of geranyl diphosphate (GPP) to bornyl diphosphate (BPP). However, the functional mechanisms of LaBPPS remain poorly understood. Here, we conducted mutational experiments based on the molecular docking results, and found that mutations at positions D356A, D360A, R497A, D501A, or E508A led to a 50- to 100-fold reduction in the activity. Deletion of region 1–58 (∆1–58) did not affect activity compared to the wild-type (WT) protein, while deletions of regions 1–74 or 59–74 (∆1–74 or ∆59–74) significantly decreased the activity. Conversely, deletion of residues 578–602 (∆578–602) dramatically increased the activity. The *LaBPPS* gene showed dramatically higher expression levels in flowers compared to other tissues (stems, leaves and roots), peaking at 8:00. Our results provide valuable insights into EO biosynthesis in lavender and suggest potential strategies for genetic engineering aimed at improving EO quality.

## 1. Introduction

Lavenders are compact, fragrant shrubs extensively cultivated for their essential oils (EOs), which are intricate blends of mono- and sesquiterpenoid alcohols, esters, oxides, and ketones [1,2,3]. The *Lavandula* genus comprises 30 recognized species, among which *Lavandula angustifolia*, *Lavandula latifolia*, and *Lavandula x intermedia*—a natural hybrid of *Lavandula latifolia* and *Lavandula angustifolia*—hold significant economic value [1,2,4]. The highest-quality EOs are extracted from the flowering tops of *Lavandula angustifolia*, commonly referred to as ‘true lavender’, renowned for its distinctive aroma and esteemed since antiquity. Lavender EOs have broad applications in the cosmetics, hygiene, and alternative medicine sectors [5,6,7,8,9]. For instance, EOs with high camphor concentrations are used in inhalants to alleviate respiratory ailments such as coughs and colds, as well as in liniments and balms for topical pain relief [10,11,12,13]. Additionally, camphor has been explored as a radiosensitizing agent, intended to improve tumor oxygenation before radiotherapy [1,2,6,14,15].

The quality of lavender EOs is largely determined by their monoterpene profile, primarily consisting of linalool, linalyl acetate, borneol, camphor, and 1,8-cineole [16,17,18,19]. Although monoterpenes exhibit significant structural diversity, they all originate from two fundamental five-carbon precursors: isopentenyl pyrophosphate and its isomer, dimethylallyl pyrophosphate [20,21]. The enzymatic activity of prenyl transferases facilitates the condensation of these precursors, generating geranyl pyrophosphate and farnesyl pyrophosphate, which serve as key intermediates for the biosynthesis of monoterpenes and sesquiterpenes, respectively [4,22,23]. Monoterpene synthases, such as bornyl diphosphate synthase, subsequently catalyze the removal of the pyrophosphate group from these linear precursors, leading to the formation of a diverse array of cyclic, linear, hydroxylated, or hydrocarbon monoterpenes [22,24,25]. Within the EO biosynthetic pathway, borneol and camphor are synthesized through the formation of bornyl diphosphate (BPP) from geranyl diphosphate (GPP), a reaction catalyzed by bornyl diphosphate synthase (BPPS) [26,27]. BPP serves as a substrate for the production of borneol, which is then oxidized to camphor by borneol dehydrogenase [26,27]. Notably, *Lavandula angustifolia* BPPS (LaBPPS) plays a pivotal role in the biosynthesis of BPP. Therefore, considerable research has focused on understanding the catalytic mechanisms of LaBPPS to gain insights into EO specificity and improve EO quality. However, the structural and mechanistic details of LaBPPS are still not understood.

Herein, we used AlphaFold2 to predict the three-dimensional structure of LaBPPS. The hydrodynamic radius of LaBPPS was determined to be 5.9 ± 0.3 nm. Based on the molecular docking results, mutational experiments were performed. We found that substitutions at D356A, D360A, R497A, D501A, or E508A led to a 50- to 100-fold reduction in enzyme activity. The activity of ∆1–58 was indistinguishable from that of the wild-type (WT) protein. In contrast, ∆1–74 or ∆59–74 caused a significant reduction in the activity compared to the WT protein. Of note, ∆578–602 resulted in a dramatic increase in the activity. The expression level of the *LaBPPS* gene was the highest in the flower among these tissues (flower, leaf, stem, and root), with a peak of expression at 8:00. These findings provide insight into the functional mechanisms of LaBPPS in lavender, contributing valuable knowledge for enhancing the quality of lavender essential oils through genetic engineering, which could have implications for the development of cosmetic, personal care, and alternative medicinal products.

## 2. Materials and Methods

### 2.1. Bioinformatics Analysis

The predicted amino acid sequence of LaBPPS (UniProt accession number A0A185NWC6) was analyzed using the ProtParam [28,29] to predict its chemical properties and physicochemical parameters. Codon optimization of all gene sequences was performed following a previously described method [30,31,32,33].

### 2.2. Protein Constructs, Expression, and Purification

The construction, expression, and purification of LaBPPS were carried out as previously described [26,27]. *E. coli* strain Rosetta (DE3) pLysS cells (Novagen, Darmstadt, Germany) were transformed with pHXGWA carrying LaBPPS via heat shock. Heterologous protein production was induced by adding 0.5 mM IPTG (isopropyl-β-d-thiogalactopyranoside) and carried out for 14 h at 16 °C and 160 rpm in terrific broth supplemented with 0.5% glycerol, 250 mM D-sorbitol, and 2.5 mM betaine. After the bacteria were harvested by centrifugation, cell disruption was performed by incubating in native binding buffer (50 mM NaH_2_PO_4_, 500 mM NaCl, 20 mM imidazole, 5% glycerol, 5 mM DTT, pH 8.0), with the addition of 0.5 mg/mL lysozyme, followed by sonication. The clarified lysate was then subjected to purification using Talon metal affinity resin (Clontech), following the manufacturer’s protocol. The resin-bound protein was incubated overnight at 4 °C in 200 mL of native binding buffer containing 10 units of thrombin. The target protein was subsequently recovered the following day by filtration.

### 2.3. Dynamic Light Scattering (DLS) Experiments

To investigate the oligomeric state of LaBPPS, the protein diameter was measured using dynamic light scattering (DLS) with a Dynapro DLS instrument (Malvern Zetasizer, Malvern, UK), following a previously described method with minor modifications [34]. LaBPPS was concentrated to approximately 2.2 mg/mL and then centrifuged at 18,000 rpm for 5 min. The protein sample was subsequently loaded into a 1 cm path length cuvette. Data acquisition involved conducting 30 runs, with an equilibration time of 120 s for each measurement. The resulting DLS data were analyzed using Zetasizer software (Version 6.20), which generated regularized DLS histograms. The protein diameter was monitored continuously throughout the analysis.

### 2.4. Structure Prediction and Quality Assessment of LaBPPS

The three-dimensional (3D) structure of LaBPPS was predicted using the AlphaFold2 program [35,36]. The LaBPPS sequence was obtained from the UniProt database under accession ID A0A185NWC6. Structural visualizations were generated using PyMOL 2.3.4 (https://www.pymol.org/2/) URL (accessed on 13 January 2025).

To validate the tertiary structures, the PDBsum tool [37,38,39] was used to generate a Ramachandran plot for LaBPPS. This tool is instrumental in assessing and validating the quality of protein structures by identifying geometric errors and ensuring the accuracy of the models. Additionally, the Ramachandran plot evaluates the stereochemical properties of the structure. The plot illustrates the dihedral angle orientations of the amino acid residues within the protein, indicating the permissible regions for residue positioning and identifying areas where certain orientations are sterically disallowed.

### 2.5. Enzymatic Activity Assays

The enzymatic activity of LaBPPS was assessed using a previously described method [26,27]. Assays were conducted in a final reaction volume of 500 μL containing 15–70 μg of purified recombinant protein, a buffer consisting of 25 mM Tris-HCl (pH 7.5), 10% glycerol, 1 mM DTT, and 1 mg/mL BSA, along with cofactors (10 mM MgCl_2_ and 1 mM MnCl_2_). The reaction was initiated by the addition of 50 μM substrate, and the mixture was overlaid with 500 μL hexane, which contained 2 mg/L methyl undecanoate as an internal standard. After a 2 h incubation at 30 °C, the mixture was vigorously mixed, and the hexane phase was collected, concentrated under a nitrogen stream, and analyzed by GC-MS. Negative controls were carried out using the purified protein without the expression vector. The Michaelis constant (*K_m_*) and the catalytic constant (*K_cat_*) were calculated using Hanes–Woolf plots.

### 2.6. GC-MS Analysis

Gas chromatography–mass spectrometry (GC-MS) analysis was performed using an Agilent 6850 gas chromatograph coupled with an Agilent 5973 ion trap mass detector, following a previously established protocol with slight modifications [26,27]. The system employed a 30 m × 0.25 mm apolar capillary column (DB5). The injector and detector temperatures were set at 250 °C, with helium as the carrier gas at a flow rate of 1.0 mL/min. The temperature program for the oven began with an initial hold at 60 °C for 4 min, followed by a ramp of 4 °C/min until the final temperature of 240 °C was reached, which was then maintained for an additional 5 min. A 2 μL sample was injected in splitless mode. During GC-MS analysis, data processing was conducted using Agilent MassHunter software, MassHunter Acquisition B.10.0 (Santa Clara, CA 95051, USA) to perform peak integration, baseline correction, and spectral deconvolution. Compound identification was achieved by matching spectra against the NIST/Wiley mass spectral libraries, applying a minimum match factor threshold of 80%. Quantitation was carried out using externally derived calibration curves for targeted analysis, whereas peak area normalization was employed for untargeted approaches. The processed data were subsequently exported for additional statistical evaluation. Metabolite identification was based on a comparison of GC-MS data with those of known reference compounds.

### 2.7. Molecular Docking

Molecular docking of the substrate GPP (geranyl diphosphate) to the protein binding site was performed using AutoDockTools 1.5.7 and AutoDock 4.2.6 [40,41,42,43], with the structural model generated by AlphaFold2. The Genetic Algorithm (GA) parameters were set to include 1000 runs and a population size of 200. The maximum number of generations and evaluations were configured at 30,000 and 3,000,000, respectively. After the docking procedure, the results were analyzed, and the interactions between LaBPPS and GPP were visualized using PyMOL v2.3.4 (https://www.pymol.org/2/) URL (accessed on 13 January 2025). The binding energy, calculated as a measure of the docking process, was found to be −5.79 kcal/mol, which is significantly lower than the threshold of −1.2 kcal/mol, confirming the reliability of the docking results. In molecular docking, the binding energy (typically expressed in kcal/mol) reflects the affinity of a ligand for its target protein. More negative values indicate stronger binding interactions, implying greater complex stability and a higher likelihood of functional modulation. This parameter serves as a key computational metric for identifying potential bioactive compounds worthy of further investigation.

### 2.8. Enzymatic Activity Assay for Site-Directed Mutagenesis Proteins

Primers for site-directed mutagenesis of the target protein were designed in-house (Figures S4 and S5; Table 1) and then synthesized by Shanghai Sangon Biotechnology (Shanghai, China). Polymerase chain reactions (PCR) were carried out using Q5 polymerase from New England Biolabs (NEB). DNA sequencing was performed by Shanghai Sangon Biotechnology to confirm the accuracy of the cloning sites. The procedures for expressing and purifying the mutated proteins followed the same protocol as for wild-type (WT) LaBPPS. Additionally, the enzymatic activities of the mutated proteins were measured under conditions identical to those used for WT (wild-type) LaBPPS.

### 2.9. LaBPPS Expression Profiles Analyzed Using RT-qPCR

The expression levels of *LaBPPS* were quantified using real-time quantitative polymerase chain reaction (RT-qPCR) with PowerUp SYBR Green Master Mix (Applied Biosystems). Total RNA was isolated employing the Universal Plant Total RNA Extraction Kit (Bioteke, Beijing, China) in accordance with the manufacturer’s protocol. Complementary DNA (cDNA) was then generated from the RNA samples using the PrimeScript 1st Strand cDNA Synthesis Kit (Takara, Kyoto, Japan). The primers used in this study are listed in Table S2. RT-qPCR amplification was carried out on an Applied Biosystems QuantStudio 5 system. For data processing, the 2^−ΔΔCT^ method [44,45,46,47,48] was applied, and relative expression levels were calculated and displayed as log_2_-transformed values in histograms. The reference gene beta-actin was used for normalization, and a positive control incorporating beta-actin was also analyzed.

### 2.10. Statistical Analysis

All experiments were conducted at least in triplicate. The data were expressed as mean ± SD. Statistical analysis was conducted using Origin 8.5, Microsoft Excel 2013, and SPSS 19.0. In all statistical evaluations, *p* < 0.05 was considered statistically significant, and *p* < 0.01 was considered highly statistically significant.

## 3. Results

### 3.1. Bioinformatics Analysis

**Figure 1:**
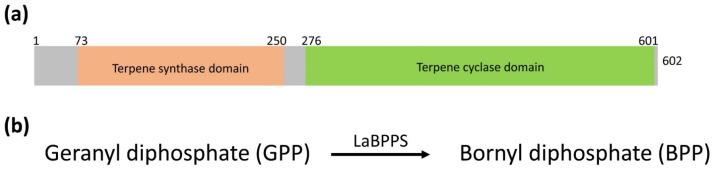
**The organization of LaBPPS clusters and the reaction.** (**a**) A schematic representation of LaBPPS. (**b**) The chemical equation describing the bornyl diphosphate synthase reaction.

*Lavandula angustifolia* bornyl diphosphate synthase (LaBPPS) possesses two conserved domains: the terpene synthase domain (aa 73–250) and the terpene cyclase domain (aa 276–601) (Figure 1a and Figure S1). LaBPPS catalyzes the conversion of geranyl diphosphate (GPP) to bornyl diphosphate (Figure 1b). The LaBPPS sequence was retrieved from the UniProt database (accession number A0A185NWC6). The molecular weight of LaBPPS is estimated to be approximately 70.67 kDa, with the molecular formula C_3162_H_4889_N_841_O_933_S_33_ and an isoelectric point (pI) of 5.16.

The effectiveness of codon optimization was evaluated by analyzing the codon adaptation index (CAI) and GC content. The optimized CAI value for LaBPPS was found to be 81.6% (Table 2). The optimal GC content range typically spans from 30% to 70%, and the optimized GC content for LaBPPS was measured at 51.9%, which falls within this optimal range (Table 2).

### 3.2. Prediction and Quality Assessment of LaBPPS Structure

**Figure 2:**
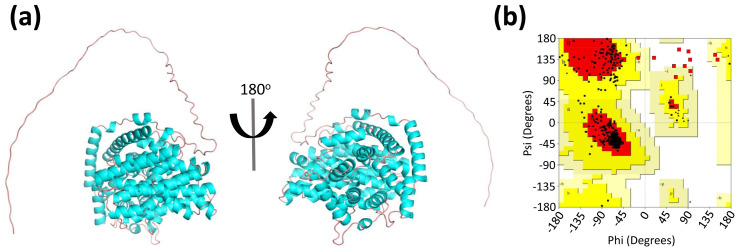
**Prediction and quality assessment of LaBPPS structural model.** (**a**) The three-dimensional (3D) structure of LaBPPS was predicted using AlphaFold2. (**b**) Structural validation of LaBPPS was performed through the Ramachandran Plot analysis. The most favored regions are indicated in red, while regions with less favorable conformations are shown in progressively lighter shades.

The three-dimensional (3D) structure of LaBPPS was predicted using AlphaFold2 [35,36,49] (Figure 2a, Figures S2 and S3). In contrast to the homology modeling methods employed in previous studies, this advanced algorithm utilizes deep learning techniques to predict protein structures with improved accuracy and reliability.

To evaluate the quality of the predicted LaBPPS structural model, the Ramachandran plot was generated to assess whether the dihedral angles of the protein backbone fell within favorable regions, thereby confirming the plausibility of the protein conformation (Figure 2b). The LaBPPS structure exhibited 89.0% of residues in the most favored region, 9.0% in the additionally allowed region, 1.1% in the generously allowed region, and 0.9% in the disallowed region (Figure 2b; Table 3). Furthermore, the counts for terminal residues, glycine residues, and proline residues were 2, 26, and 18, respectively (Figure 2b, Table 3). These findings suggested that the LaBPPS structural model is of high quality.

### 3.3. Characterization of LaBPPS by Dynamic Light Scattering

**Figure 3:**
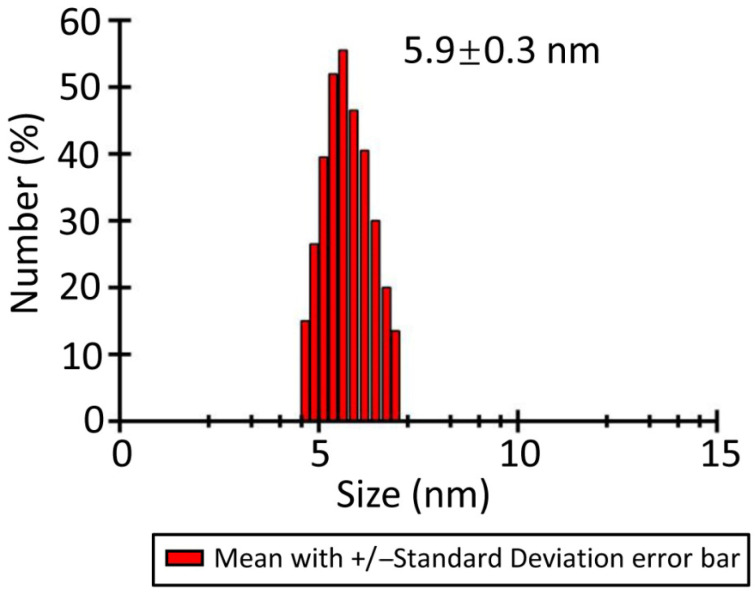
**Dynamic light scattering (DLS) spectrum of LaBPPS.** The analysis revealed that LaBPPS has a hydrodynamic radius of 5.9 ± 0.3 nm.

To further examine the oligomeric state of LaBPPS, dynamic light scattering (DLS) experiments were conducted to determine its hydrodynamic radius after centrifugation. The results showed a hydrodynamic radius of 5.9 ± 0.3 nm for LaBPPS (Figure 3), indicating that LaBPPS exists in its monomeric form.

### 3.4. The Predicted Ligand-Binding Sites of LaBPPS-GPP Complex

**Figure 4:**
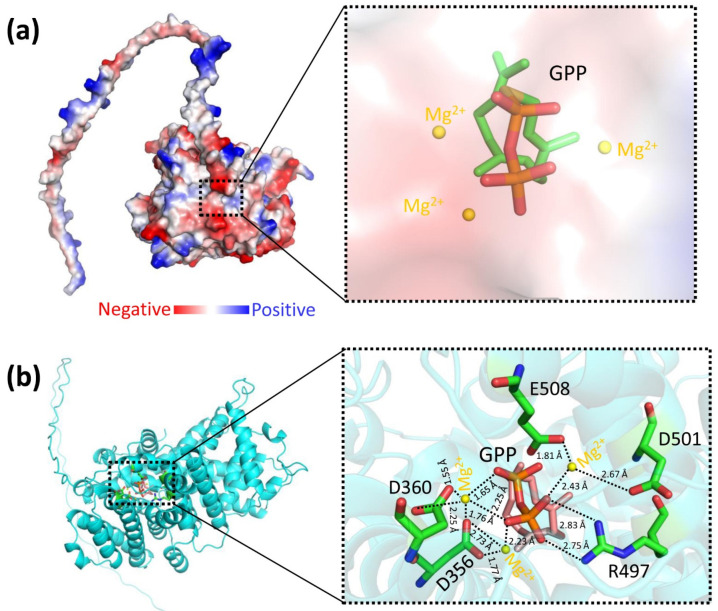
**The structural model of LaBPPS-GPP binary complex.** The substrate GPP is depicted in stick representation, and magnesium ion (Mg^2+^) is shown as yellow spheres. (**a**) The surface representation of the model, with color coding indicating charge distribution: red for negative charges, blue for positive charges, and white for neutral charges. (**b**) The ribbon representation of the model, with a close-up view of the active sites on the right. Polar and charged interactions are represented by dashed lines.

The structural prediction of LaBPPS using AlphaFold2 [35,36,49] demonstrated high reliability (Figure 2; Table 3). The structural model included both the terpene synthase and terpene cyclase domains (Figure 1 and Figure S1). Based on this model, molecular docking was performed using AutoDock software, AutoDock 4.2.6 (La Jolla, CA 92037, USA) to generate a protein–substrate complex (Figure 4). The binding energy, which served as an indicator of docking success, was calculated to be −5.79 kcal/mol, significantly exceeding the threshold of −1.2 kcal/mol, thereby confirming the validity of the docking results. In the predicted protein–substrate complex, the substrate geranyl diphosphate (GPP) was positioned securely within the binding pocket, with electrostatic interactions involving magnesium ions (Mg^2+^), the phosphate group of GPP, and the side chains of aspartic acid and glutamic acid facilitating the binding (Figure 4).

**Figure 5:**
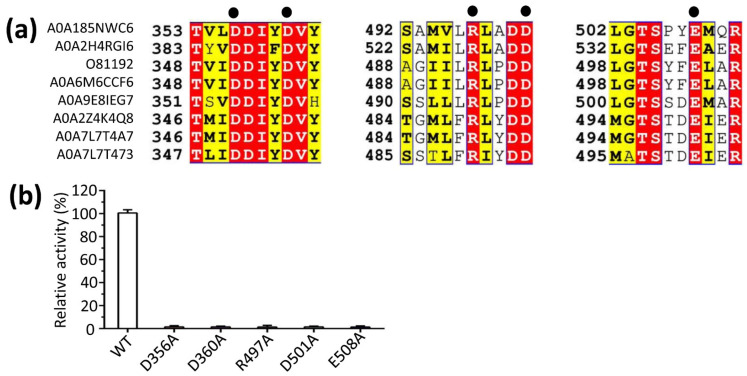
**Enzymatic characterization of LaBPPS to assess the bornyl diphosphate synthase activity.** (**a**) Sequence alignment of conserved residues from various species. A0A185NWC6, *Lavandula angustifolia* subsp. *angustifolia*; A0A2H4RGI6, *Phyla dulcis* (*Aztec sweet herb*, *Lippia dulcis*); O81192, *Salvia officinalis* (*Sage*); A0A6M6CCF6, *Salvia officinalis* (*Sage*); A0A9E8IEG7, *Artemisia annua* (*Sweet wormwood*); A0A2Z4K4Q8, *Wurfbainia villosa*; A0A7L7T4A7, *Wurfbainia villosa*; A0A7L7T473, *Wurfbainia longiligularis*. (**b**) The activities of wild-type (WT) LaBPPS and specific mutants were quantified. Mutants D356A, D360A, R497A, D501A, or E508A exhibited a reduction in the activity ranging from 50- to 100-fold. The activity of WT LaBPPS was set to 100%.

The results of mutational experiments showed that substituting alanine for the amino acids D356, D360, R497, D501, or E508 resulted in a 50- to 100-fold reduction in enzymatic activity (Figure 5). This suggested that these negatively charged residues are strategically positioned to interact electrostatically with the positively charged magnesium ion (Mg^2+^), thereby stabilizing and neutralizing the phosphate group of the substrate GPP. This evidence underscores the critical role of these conserved negatively charged residues (D356, D360, R497, D501, and E508) in maintaining the bornyl diphosphate synthase activity of LaBPPS.

### 3.5. The N- and C-Terminus Regulated the Catalytic Activity of LaBPPS

**Figure 6:**
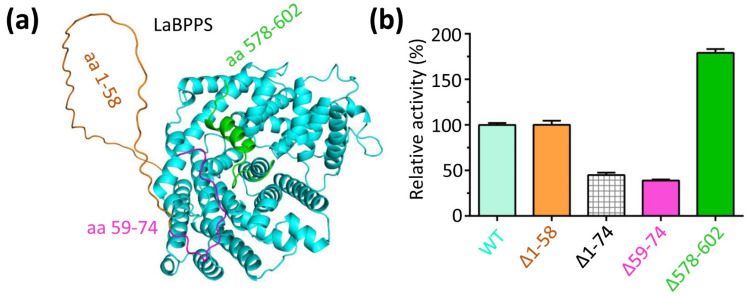
**N- and C-terminus regulated the activity of LaBPPS.** (**a**) The regions spanning residues 1–58, 59–74, and 578–602 are colored in orange, magenta, and green, respectively. (**b**) Deletion of region 1–58 (∆1–58) did not affect the activity, which remained identical to that of the wild-type (WT) protein. In contrast, deletions of region 1–74 or 59–74 (∆1–74 or ∆59–74) resulted in a significant reduction in the activity compared to the WT protein. However, deletion of 578–602 (∆578–602) led to a substantial increase in the activity. The activity of WT LaBPPS was set to 100%.

The N-terminal and C-terminal regions are located at the periphery of the catalytic pocket (Figure 6a). Deletion of amino acids 578–602 from the C-terminus (∆578–602) resulted in a significant increase in enzyme activity (Figure 6b). This suggested that the 578–602 region of the C-terminus undergoes structural changes that may hinder substrate (GPP) binding to the catalytic cavity, thereby inhibiting the catalytic reaction. These findings indicated that the flexibility of region 578–602 plays a crucial role in modulating the activity of LaBPPS.

For the N-terminal deletion, the activity of the ∆1–58 mutant was comparable to that of the full-length protein (Figure 6b), indicating that the region from residues 1 to 58 is not critical for the activity of LaBPPS. To further investigate the functional mechanisms, additional truncations of LaBPPS were performed. Deletion of 1–74 (∆1–74) resulted in a significant decrease in activity (Figure 6b). In an effort to further explore this, the activity of the ∆59–74 mutant was assessed, indicating a dramatic reduction in activity (Figure 6b). These findings suggested that the 59–74 region, rather than the 1–58 region, plays a key role in the catalytic activity of LaBPPS.

On the other hand, the Michaelis constant (*K_m_*) values for wild-type (WT) LaBPPS and ∆1–58 were 29.37 μM and 28.12 μM, respectively, both of which were significantly lower than those of ∆1–74 (41.72 μM) and ∆59–74 (38.49 μM), but considerably higher than the *K_m_* value of ∆578–602 (14.83 μM) (Table 4). In contrast, the catalytic constant (*K_cat_*) values for WT LaBPPS and ∆1–58 (6.23 min^−1^ for WT and 6.78 min^−1^ for ∆1–58) were notably higher than those of ∆1–74 (3.19 min^−1^) and ∆59–74 (3.37 min^−1^) (Table 4), yet lower than the value for ∆578–602 (11.71 min^−1^) (Table 4). These results suggest that the ∆578–602 mutant exhibits a higher substrate affinity and a faster reaction rate than the WT protein. This implies that the 578–602 region may partially obstruct substrate GPP binding to the catalytic cavity, thereby inhibiting the catalytic reaction.

### 3.6. Spatial and Temporal Regulation Patterns of LaBPPS Gene

**Figure 7:**
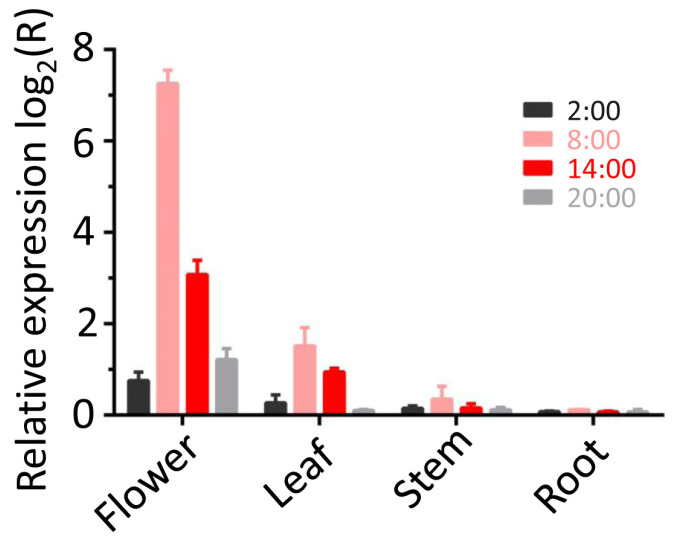
***LaBPPS*** **gene expression profiles in different tissues (flower, leaf, stem, and root) during a 24 h day/night cycle.** *LaBPPS* gene expression was significantly higher in flower compared to leaf, stem, and root, peaking at 8:00. The relative expression was assessed by RT-qPCR using beta-actin as a reference gene. The expression ratios are presented as log_2_ values, where a ratio greater than zero indicates an up-regulation.

To assess *LaBPPS* expression patterns across different tissues and time points, real-time quantitative polymerase chain reaction (RT-qPCR) was conducted. The results revealed markedly higher transcript levels in flowers compared to leaves, stems, and roots, with peak expression occurring at 8:00 (Figure 7). At this time point, flowers exhibited the highest expression levels (152.9-fold), substantially surpassing levels in leaves (2.9-fold), stems (1.3-fold), and roots (1.1-fold). On the other hand, temporal profiling in flowers showed maximal expression at 8:00 (152.9-fold), followed by 14:00 (8.5-fold), while 20:00 and 2:00 displayed lower expression (2.3-fold and 1.7-fold, respectively) (Figure 7). These results identify flowers as the dominant site of *LaBPPS* activity. The observed tissue-specific and diurnal expression patterns suggest spatially and temporally regulated terpene synthesis, indicating transcriptional control over terpenoid biosynthesis.

To further investigate the spatiotemporal dynamics of gene expression, we analyzed *LaBPPS*-derived metabolites using gas chromatography–mass spectrometry (GC-MS). We found that the highest metabolite accumulation was detected in the flower at 8:00 among these conditions (Table 5). The highest yield of metabolites was observed in the flower compared to the corresponding yields from the other tissues (leaf, stem and root) (Table 5). Notably, metabolite production at 8:00 significantly surpassed yields assessed at other time points (2:00, 14:00, and 20:00) (Table 5). This pattern aligns with the transcriptional activity revealed by the RT-qPCR results, confirming coordinated expression and metabolic output of the *LaBPPS* gene.

## 4. Discussion

In this study, AlphaFold2 was employed to predict the three-dimensional structure of LaBPPS. The hydrodynamic radius of LaBPPS was measured to be 5.9 ± 0.3 nm. According to the molecular docking results, mutational experiments were performed. Substitutions at D356A, D360A, R497A, D501A, or E508A resulted in a 50- to 100-fold reduction in enzymatic activity. The activity of the ∆1–58 mutant was found to be equivalent to that of the wild-type (WT) protein. Deletion of regions 1–74 or 59–74 (∆1–74 or ∆59–74) significantly diminished activity compared to the WT protein, while deletion of 578–602 (∆578–602) led to a marked increase in activity. The expression level of the *LaBPPS* gene was significantly higher in the flower than in the stem and root, and leaf, with a peak at 8:00. These findings provide critical insights into the functional mechanisms of LaBPPS and offer potential strategies for improving the quality of lavender essential oils through genetic engineering.

Due to the inability to obtain crystals of LaBPPS, we pursued a more detailed investigation into its functional mechanism. To aid in this analysis, we utilized SWISS-MODEL [50,51,52,53] to identify structural homologs of LaBPPS (Table S1). Our search revealed that LaBPPS shared amino acid sequence identities of 53.83%, 53.64%, 53.13%, 50.47%, and 45.81% with terpene synthases from *Mentha spicata*, *Salvia officinalis*, *Salvia fruticosa*, *Thymus vulgaris*, and *Citrus sinensis*, respectively (Table S1). These results provided valuable insights into the structural and functional mechanisms of LaBPPS in lavender.

Essential oils (EOs) from the genus lavender primarily consist of a limited number of ‘regular’ monoterpenes and their derivatives [16,54]. For instance, the economically valuable EOs from *Lavandula angustifolia* and *Lavandula x intermedia* species are rich in compounds such as linalool, linalool acetate, and 1,8-cineole [55]. In contrast, emerging synthetic biology platforms have shown great promise as an alternative method for producing monoterpenoids [14,56,57]. By engineering microbial biosynthetic pathways, sustainable and efficient production systems can be established. Notable successes include the microbial production of geraniol, limonene, linalool, (+)-borneol, citronellol, and nerol. (−)-Borneol is primarily derived from geranyl diphosphate (GPP), which is synthesized from the two common C_5_ precursors, isopentenyl diphosphate and dimethylallyl diphosphate, through either the cytoplasmic mevalonate (MVA) pathway or the plastidic 2-C-methyl-D-erythritol-4-phosphate (MEP) pathway [10,17,55,56,58].

In conclusion, our study presents a novel approach for the thorough investigation of the complex functional mechanisms of LaBPPS in lavender, with the aim of enhancing the quality of lavender EOs.

## Figures and Tables

**Table 1:** Primers used for generating site-directed mutants of *LaBPPS*.

Primers	Primer Sequence (5′-3′)
D356A (F)	GTCCTG**gca**GACATCTACGATG
D356A (R)	GTAGATGTC**tgc**CAGGACCGTGATC
D360A (F)	CATCTAC**gca**GTATACGGCACCC
D360A (R)	CGTATAC**tgc**GTAGATGTCGTCCAGG
R497A (F)	GGTTCTG**gca**CTGGCAGATGAC
R497A (R)	CTGCCAG**tgc**CAGAACCATG
D501A (F)	CTGGCAGAT**gca**CTGGGCACCTCCC
D501A (R)	GGTGCCCAG**tgc**ATCTGCCAGACGC
E508A (F)	CCCGTAC**gca**ATGCAACGTGGTG
E508A (R)	GTTGCAT**tgc**GTACGGGGAGGTG

Note: Mutagenic regions of the sequence are shown in underlined, and bold.

**Table 2:** Codon optimization of *LaBPPS*.

Codon Optimization	Codon Adaptation Index (CAI) Value	GC Content Value
Before codon optimization	46.2%	43.0%
After codon optimization	81.6%	51.9%

**Table 3:** Ramchandran plot analysis of LaBPPS model using PDBsum.

Residues	Residues in Most Favored Regions		Residues in Additional Allowed Regions		Residues in Generously Allowed Regions		Residues in Disallowed Regions	
Residual Properties	Number of residues	Total % of residues	Number of residues	Total % of residues	Number of residues	Total % of residues	Number of residues	Total % of residues
LaBPPS	495	89.0	50	9.0	6	1.1	5	0.9

Note: Number of end-residues (excl. Gly and Pro): 2; number of glycine residues (shown as triangles): 26; number of proline residues: 18.

**Table 4:** Kinetic parameters of different LaBPPS constructs.

Constructs	*K_m_* (μM)	*K_cat_* (min^−1^)
WT (wild-type)	29.37 ± 1.21	6.23 ± 0.47
∆1–58	28.12 ± 1.75	6.78 ± 0.35
∆1–74	41.72 ± 2.15	3.19 ± 0.12
∆59–74	38.49 ± 2.58	3.37 ± 0.42
∆578–602	14.83 ± 1.09	11.71 ± 0.86

Note: Kinetic parameters were determined using Hanes–Woolf plots.

**Table 5:** Analysis of metabolite derived from LaBPPS.

Tissues	Time Points	Metabolite (Quantity μg/g Dry Tissue)
Flower	2:00	1.29 ± 0.56
	8:00	239.53 ± 6.47
	14:00	13.27 ± 1.14
	20:00	3.47 ± 0.83
Leaf	2:00	0.38 ± 0.02
	8:00	8.49 ± 0.92
	14:00	1.95 ± 0.73
	20:00	0.09 ± 0.01
Stem	2:00	0.11 ± 0.06
	8:00	0.95 ± 0.06
	14:00	0.25 ± 0.03
	20:00	0.13 ± 0.09
Root	2:00	0.08 ± 0.02
	8:00	0.38 ± 0.01
	14:00	0.06 ± 0.01
	20:00	0.05 ± 0.02

## Data Availability

The data presented in this study are available in this article (and Supplementary Materials), and further enquiries can be directed to the corresponding authors.

## Supplementary Materials

The following supporting information can be downloaded at https://www.mdpi.com/article/10.3390/cimb47070517/s1.
