# Supplementary material for: Mechanistic Insights into the Bornyl Diphosphate Synthase from Lavandula angustifolia
Source: Curr Issues Mol Biol. 2025 Jul 4;47(7):517. doi: 10.3390/cimb47070517 (PMC12293770; doi:10.3390/cimb47070517)
Supplement: Supplementary file 1 [file cimb-47-00517-s001.zip › cimb-3681631-supplementary.pdf]

Supplementary Material

Mechanistic insights into the bornyl diphosphate synthase from *Lavandula angustifolia*

Dafeng Liu[1,*], Na Li[1], Feng Yu[1], Yanyan Du[1], Hongjun Song[2] and Wenshuang Yao[2]

[1]Xinjiang Key Laboratory of Lavender Conservation and Utilization, College of Biological Sciences and Technology, Yili Normal University, Yining 835000, Xinjiang, China;

[2]School of Life Sciences, Xiamen University, Xiamen 361102, Fujian, China.

[*]Correspondence: dafeli@sina.cn or dafeli-dafeli@hotmail.com

Figure S1.

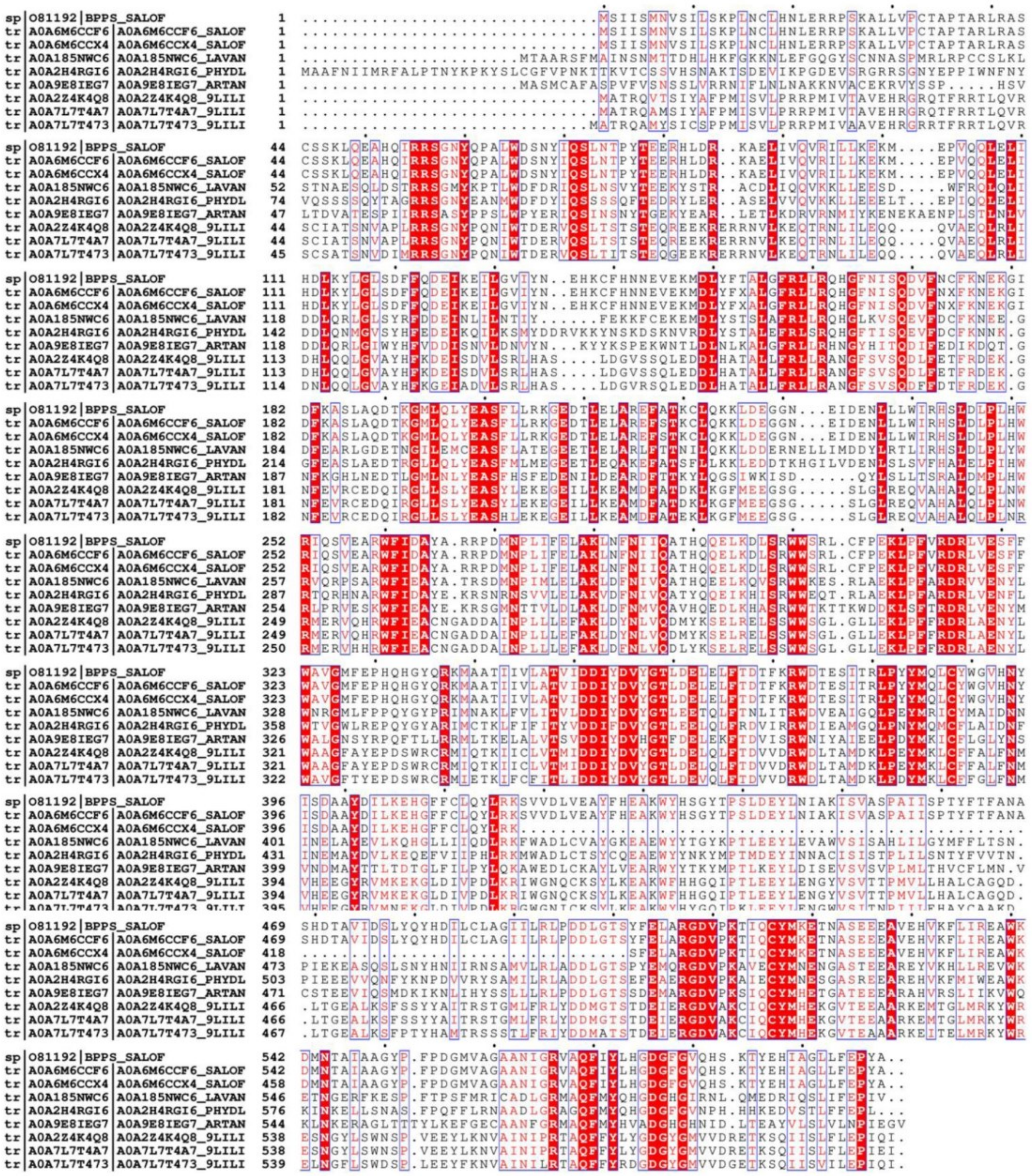

Figure S1. Sequence alignments of bornyl diphosphate synthases. The ClustalW default color scheme is employed, wherein conserved amino acids are depicted with more intense colors compared to non-conserved ones. The alignment includes the following reference proteins: O81192, *Salvia officinalis* (Sage); A0A6M6CCF6, *Salvia officinalis* (Sage); A0A6M6CCX4, *Salvia officinalis* (Sage); A0A185NWC6, *Lavandula angustifolia subsp. Angustifolia*; A0A2H4RGI6, *Phyla dulcis* (Aztec sweet herb, Lippia dulcis); A0A9E8IEG7, *Artemisia annua* (Sweet wormwood); A0A2Z4K4Q8, *Wurfbainia villosa*; A0A7L7T4A7, *Wurfbainia villosa*; A0A7L7T473, *Wurfbainia longiligularis*.

Figure S2.

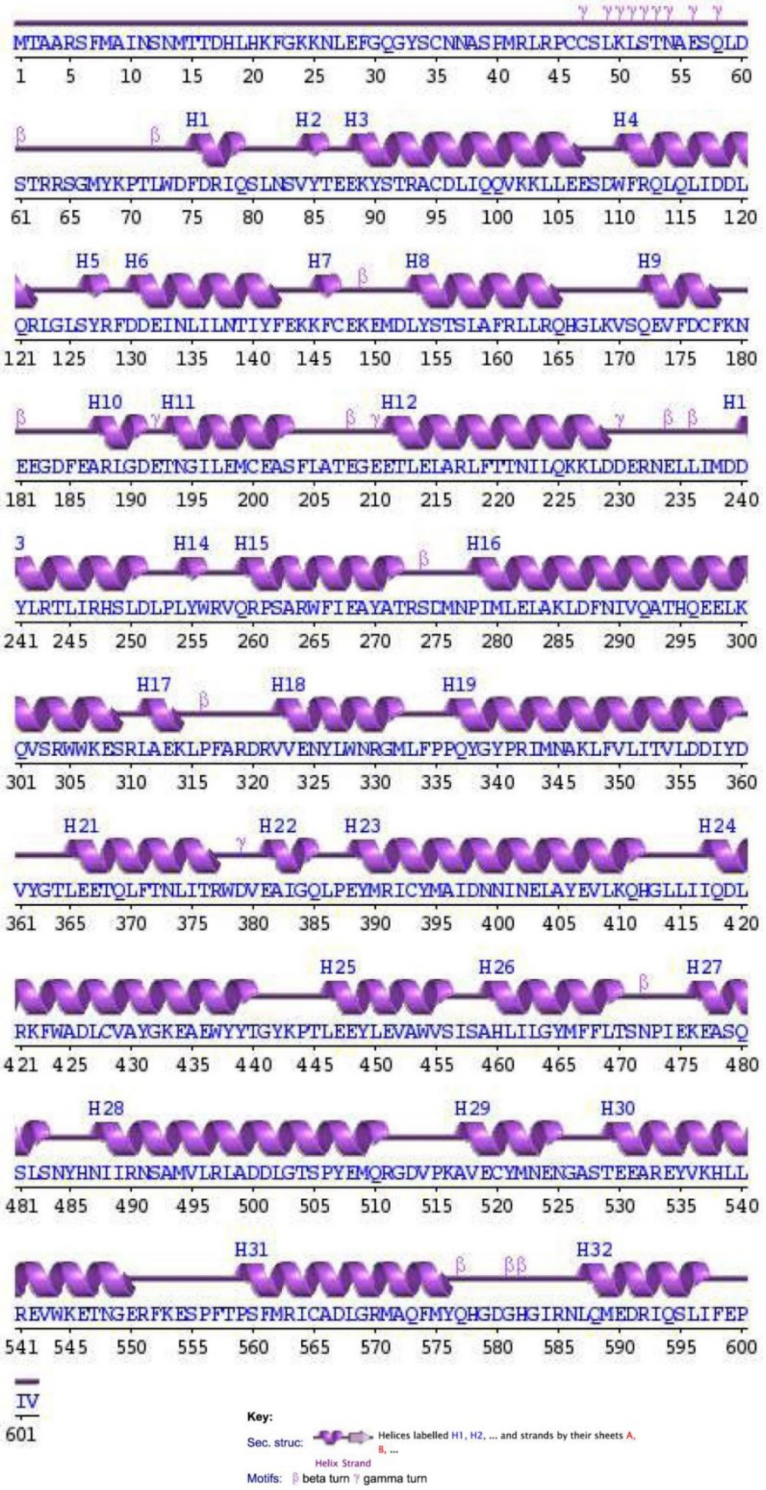

Figure S2. Secondary structure of LaBPPS.

Figure S3.

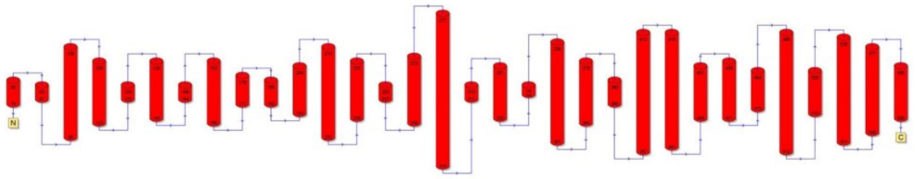

Figure S3. Secondary structure of LaBPPS.

Figure S4.

ATGACCGCAGCCCGTTCCTTTATGGCAATCAATTCTAACATGACCACTGACCATCTGCACAAATTCGGTAAAA
AGAACCTGGAGTTCGGCCAAGGCTACAGCTGCAACAACGCGTCTCCTATGCGCCTGCGCCCGTGCTGTTCC
CTGAAACTGTCTACGAACGCGGAAAGCCAGCTGGATTCTACTCGCCGTTCCGGTATGTATAAACCGACCCTG
TGGGATTTCGATCGCATCCAGAGCCTGAACTCTGTGTACACTGAAGAAAAATACTCCACGCGCGCCTGCGAC
CTGATCCAGCAGGTGAAAAAGCTGCTGGAGGAAAGCGACTGGTTCCGTCAGCTGCAACTGATCGACGACC
TGCAGCGTCTGGGTCTGTCCTACCGCTTCGATGATGAAATCAACCTGATCCTGAACACGATTTATTTTGAAAA
AAAATTCTGTGAAAAAGAGATGGACCTGTACAGCACTTCCCTGGCCTTCCGTCTGCTGCGTCAGCATGGTCT
GAAGGTTAGCCAGGAGGTCTTCGATTGCTTCAAGAACGAGGAAGGTGATTTCGAGGCTCGTCTGGGCGAT
GAAACTAATGGCATCCTGGAGATGTGTGAGGCTTCTTTCCTGGCGACCGAAGGCGAAGAGACTCTGGAGCT
GGCACGTCTGTTCACGACCAACATCCTGCAGAAGAAACTGGACGATGAACGCAACGAGCTGCTGATCATGG
ATGACTATCTGCGCACTCTGATCCGTCACTCTCTGGATCTGCCACTGTACTGGCGTGTTCAGCGTCCTAGCGC
ACGTTGGTTCATTGAGGCATACGCAACCCGTTCCGATATGAATCCTATCATGCTGGAGCTGGCTAAGCTGGAC
TTCAACATTGTTCAAGCTACCCATCAGGAAGAACTGAAACAAGTGTCCCGTTGGTGGAAAGAAAGCCGTCT
GGCGGAAAAACTGCCATTCGCGCGTGATCGTGTTGTAGAAAACTATCTGTGGAACCGTGGTATGCTGTTCCC
GCCACAGTATGGCTACCCGCGTATCATGAACGCAAAACTGTTCGTACTGATTACCGTTCTGGACGACATCTAC
GATGTTTACGGCACCCTGGAGGAGACCCAACTGTTCACCAATCTGATCACTCGTTGGGATGTCGAGGCGATT
GGTCAACTGCCAGAATACATGCGCATCTGCTACATGGCGATCGACAACAACATCAACGAGCTGGCTTACGAA
GTACTGAAACAGCACGGCCTGCTGATTATTCAAGATCTGCGCAAATTCTGGGCAGATCTGTGCGTGGCATAC
GGCAAAGAGGCAGAGTGGTACTACACCGGCTATAAGCCGACTCTGGAAGAATACCTGGAAGTCGCGTGGG
TGTCTATCTCCGCGCATCTGATTCTGGGTTACATGTTCTTCCTGACGTCCAACCCGATCGAAAAAGAGGCCTC
TCAGTCCCTGAGCAACTACCACAACATCATTCGCAACAGCGCTATGGTTCTGCGCCTGGCGGATGACCTGGG
TACCTCTCCATACGAGATGCAGCGCGGCGATGTCCCGAAGGCTGTTGAATGCTACATGAACGAAAACGGTG
CGTCCACCGAGGAAGCACGTGAATACGTGAAGCACCTGCTGCGTGAAGTCTGGAAAGAAACTAACGGCGA
GCGTTTCAAAGAATCTCCGTTCACTCCGTCCTTCATGCGCATCTGTGCAGATCTGGGTCGCATGGCACAGTTT
ATGTACCAGCATGGCGATGGTCACGGCATCCGTAATCTGCAAATGGAAGACCGTATCCAATCTCTGATCTTCG
AGCCGATTGTT

Figure S4. Gene sequence of *LaBPPS* after codon optimization

Figure S5.

MTAARSFMAINSNMTTDHLHKFGKKNLEFGQGYSCNNASPMRLRPCCSLKLSTNAESQLDSTRRSGMYKPTL
WDFDRIQSLNSVYTEEKYSTRACDLIQQVKKLLEESDWFRQLQLIDDLQRLGLSYRFDDEINLILNTIYFEKKFCEK
EMDLYSTSLAFRLLRQHGLKVSQEVFDCFKNEEGDFEARLGDETNGILEMCEASFLATEGEETLELARLFTTNILQ
KKLDDERNELLIMDDYLRTLIRHSLDLPLYWRVQRPSARWFIEAYATRSDMNPIMLELAKLDFNIVQATHQEELK
QVSRWWKESRLAEKLPFARDRVVENYLWNRGMLFPPQYGYPRIMNAKLFVLITVLDDIYDVYGTLEETQLFTN
LITRWDVEAIGQLPEYMRICYMAIDNNINELAYEVLKQHGLLIIQDLRKFWADLCVAYGKEAEWYYTGYKPTLEE
YLEVAWVSISAHLILGYMFFLTSNPIEKEASQSLSNYHNIIRNSAMVLRLADDLGTSPYEMQRGDVPKAVECYM
NENGASTEEAREYVKHLLREVWKETNGERFKESPFTPSFMRICADLGRMAQFMYQHGDGHGIRNLQMEDRI
QSLIFEPIV

Figure S5. Protein sequence of LaBPPS

Table S1.

Table S1. Search for structural homologs of LaBPPS using SWISS-MODEL

Rank	PDB code	GMQE	Identity (%)	Method	Oligo State	Ligands	Description
1	2onh	0.76	53.83	X-ray, 2.7 Å	homo-dimer	6xMN, 2xF3P, 1xBTB	4S-limonene synthase; Crystal Structure of limonene synthase with 2-fluorolinalyl diphosphate(FLPP) from *Mentha spicata*
2	1n1z	0.78	53.64	X-ray, 2.0 Å	homo-dimer	2xMG, 11xHG	(+)-bornyl diphosphate synthase; Crystal Structure of (+)-Bornyl Diphosphate Synthase from *Salvia officinalis*
3	2j5c	0.71	53.13	X-ray, 2.0 Å	monomer	None	1,8-CINEOLE SYNTHASE; Rational conversion of substrate and product specificity in a monoterpene synthase. Structural insights into the molecular basis of rapid evolution from *Salvia fruticosa*
4	5c05	0.75	50.47	X-ray, 1.6 Å	homo-dimer	None	Putative gamma-terpinene synthase; Crystal Structure of Gamma-terpinene Synthase from *Thymus vulgaris*
5	5uv0	0.72	45.81	X-ray, 2.3 Å	monomer	None	(+)-limonene synthase; Crystal Structure of (+)-Limonene Synthase from *Citrus sinensis*

Table S2.

Table S2. Primers used for RT-qPCR in this study

Genes	Primers	Primer sequence (5'-3')
Beta-actin	Forward primer	atggccgaagccgaggatattcagc
	Reverse primer	ttagaagcattttctgtgaacgatcgacgggc
LaBPPS	Forward primer	atgactgctgcaagatcattcatggcgatc
	Reverse primer	aacaataggttcaaatatcaaactttgaatgcggtcctcc
